# The most appropriate indicators of successful ovarian stimulation

**DOI:** 10.1186/s12958-024-01331-6

**Published:** 2025-01-09

**Authors:** Matheus Roque, Sesh K. Sunkara

**Affiliations:** 1 Mater Prime – Reproductive Medicine, Av. Ibirapuera, 2315 – Moema, São Paulo, 04029-200 SP Brazil; 2 King’s College London, London, UK

**Keywords:** Ovarian stimulation, IVF, Live birth, Cumulative live birth

## Abstract

Ovarian stimulation (OS) is a crucial component of clinical IVF treatment that strongly influences outcomes. As such, it is useful to understand the indicators for successful OS during IVF. As OS leads to multiple follicular recruitment, it can be quantified as number of oocytes retrieved. Optimal OS should help to maximize the number of oocytes, thus improving preclinical laboratory outcomes. Optimal preclinical outcomes should ultimately lead to clinical outcomes with maximal efficacy, safety, and cost-effectiveness. To help guide successful OS, this review details prognostic factors and appropriate endpoints for an optimal OS at each stage of the IVF cycle.

## Introduction

Ovarian stimulation (OS) is a major component of IVF treatment, and the aim of OS in IVF is to maximize success with safety and cost-effectiveness. The definition of success in IVF has evolved over the years, thanks to scientific advances and innovations. Although the most common definition is live birth per cycle, per oocyte retrieval, or per embryo transfer procedure, the development of safe and effective OS strategies and improved cryopreservation techniques such as vitrification has extended the definition of success to encompass cumulative live birth rate (CLBR) and time to live birth. CLBR is the live births resulting from embryos from one initiated cycle [1], and reflects the overall efficiency of the IVF cycle. Time to live birth should also be considered an important outcome to measure effectiveness with fertility treatments, as a short time to live birth reduces patient burden in addition to being cost-effective [2, 3].

Preclinical and laboratory outcomes following OS are as vital as the clinical outcomes, given the overwhelming evidence of an association between the two. It is therefore important to incorporate preclinical and laboratory indicators as measures of OS. Such outcomes include number of oocytes retrieved, number of mature oocytes, number of transferable embryos, and number of euploid embryos resulting from one stimulation.

Safety is an important measure of a successful OS, and avoiding iatrogenic ovarian hyperstimulation syndrome (OHSS) risk is paramount. Other safety outcomes include pregnancy outcomes with resulting pregnancies. The review will address how to define a successful OS by looking into the association of OS quantified as the number of oocytes retrieved and the various pre-clinical and clinical outcomes.

## The role of ovarian stimulation in IVF treatments

OS is an essential component of IVF treatment, as it allows the collection of multiple oocytes to increase the chances of obtaining viable embryos for transfer. The drugs used for OS include gonadotropin-releasing hormone (GnRH) agonists, GnRH antagonists, and follicle-stimulating hormone (FSH) and luteinizing hormone analogs. The choice of medication depends on various factors such as patient age, ovarian reserve, and body mass index (BMI) [4].

However, OS carries the risk of adverse effects, including OHSS, which can be mild or severe. Mild OHSS is common and usually resolves within a few days without treatment, but severe OHSS is rare and can be life-threatening. Other potential side effects of OS include ovarian torsion, infection, and psychological distress [1].

## Factors affecting outcomes of ovarian stimulation

The success of OS can be influenced by various factors, including age, BMI, ovarian reserve, and type of infertility. Older women have a lower ovarian reserve and are less likely to respond to OS, resulting in a lower number of oocytes retrieved and a lower chance of pregnancy [4]. Higher BMI is also associated with reduced ovarian response, although the exact mechanism is unclear [5].

Ovarian reserve, as assessed by serum anti-Müllerian hormone (AMH) levels and antral follicle count (AFC), is a predictor of ovarian response and success rates in IVF. Women with lower AMH levels and AFC are less likely to respond to OS, and have a lower chance of pregnancy [6]. The type of infertility, such as male factor infertility or tubal factor infertility, can also affect the success of OS [4].

Apart from these factors, lifestyle factors such as smoking, alcohol consumption, and diet can also affect OS outcomes. Smoking has been shown to decrease ovarian reserve and response to OS. The detrimental effects of smoking on ovarian function are thought to be due to the toxic chemicals in cigarettes, which damage the follicles and decrease blood flow to the ovaries [7, 8]. Similarly, heavy alcohol consumption has been associated with decreased ovarian reserve and response to OS [8]. The exact mechanism by which alcohol affects ovarian function is unclear, but it might be related to the toxic effects of alcohol on the liver, which can lead to hormonal imbalances that affect ovarian function [9].

Diet also plays a role in ovarian function and response to OS, mainly in patients with polycystic ovary syndrome. A diet high in saturated fats and low in antioxidants has been associated with impairment in the hypothalamic-pituitary-ovarian axis and response to OS [10]. Conversely, a diet high in fruits, vegetables, and whole grains has been associated with improved ovarian function [5]. The beneficial effects of a healthy diet on ovarian function are thought to be due to the high levels of antioxidants in fruits and vegetables, which can help to protect the ovaries from oxidative stress and damage [11].

Genetic factors also play a role in ovarian reserve and response to OS. Variations in genes involved in ovarian development and function, such as the *FSHR* and *AMH* genes, have been associated with differences in ovarian reserve and response to OS [12, 13]. For example, certain variations in the *FSHR* gene have been associated with a lower number of oocytes retrieved and a reduced chance of pregnancy [13]. Similarly, certain variations in the *AMH* gene have been associated with a lower AMH level and lower number of oocytes retrieved [13, 14]. Understanding the genetic factors that influence ovarian function can help to predict a woman’s response to OS and personalize her treatment plan accordingly. Genetic predispositions and clinical variants significantly influence the outcomes of ovarian stimulation. Recent studies have underscored the importance of genetic factors such as polymorphisms in the *FSH* receptor gene and variations in the *AMH* gene, which can markedly affect ovarian response to stimulation [15, 16]. Such insights are pivotal for developing personalized medicine frameworks in reproductive technologies, where treatment customization based on genetic makeup could significantly enhance clinical outcomes [17, 18].

In addition to age, BMI, ovarian reserve, and type of infertility, the POSEIDON criteria offer a nuanced approach to categorizing patients undergoing IVF based on their ovarian reserve and response to stimulation. This stratification is crucial as it recognizes the heterogeneity among patients with ‘poor ovarian response’. By delineating four distinct groups, POSEIDON allows for more personalized treatment approaches. Groups 1 and 2 consist of younger women (≤ 35 years) and older women (> 35 years), respectively, both with adequate ovarian reserve but with unexpected poor or suboptimal response to ovarian stimulation. Groups 3 and 4, on the other hand, include women with diminished ovarian reserve, again stratified by age. This classification aids in identifying patients who might benefit from modified stimulation protocols or adjunct treatments to improve their reproductive outcomes [19, 20]. By utilizing this stratification, clinicians can more accurately predict ovarian response and tailor stimulation protocols accordingly [21, 22].

## Safety outcomes of ovarian stimulation

Safety outcomes are an important consideration in OS, and avoiding iatrogenic OHSS is a paramount concern. OHSS is a potentially life-threatening condition that occurs when there is an excessive ovarian response to stimulation [1].

Various strategies have been developed to minimize the risk of OHSS, including using lower doses of medication, using GnRH agonist triggering instead of human chorionic gonadotropin (hCG) triggering, and using freeze-all cycles. GnRH agonist triggering involves using a GnRH agonist instead of hCG to trigger final oocyte maturation and ovulation. This approach reduces the risk of OHSS by inducing a more physiologic final maturation of the oocytes, which is less likely to cause a severe ovarian response [23, 24]. In addition, GnRH agonist triggering associated to a modified luteal-phase support has been shown to reduce the risk of early pregnancy loss and increase live birth rates in women at high risk of OHSS [25].

Another strategy to prevent OHSS is to use freeze-all cycles. This involves collecting and fertilizing the oocytes, but not transferring any embryos to the uterus immediately. Instead, the embryos are frozen and transferred later, once the risk of OHSS has subsided. Freeze-all cycles have been shown to reduce the risk of OHSS in women at high risk, while maintaining similar success rates to fresh embryo transfer cycles [4, 26]. Freeze-all cycles, also known as deferred embryo transfer (DET) cycles, involve freezing all embryos obtained during OS and transferring them in a subsequent cycle. This approach allows for the use of controlled OS without the risk of OHSS, as the embryos are not transferred in the same cycle. Freeze-all cycles may also improve pregnancy outcomes by allowing for endometrial preparation before embryo transfer, which can result in a more receptive endometrium [4].

In addition to these strategies, the use of personalized OS protocols based on individual patient characteristics may also help to prevent OHSS. A recent study demonstrated that individualized gonadotropin dosing based on patient characteristics such as age and ovarian reserve resulted in a lower incidence of OHSS compared to standard protocols. Personalized protocols may also improve the efficiency of OS and increase success rates [27].

Overall, the prevention of OHSS is a critical component of safe and successful OS in IVF treatments. Strategies such as using GnRH agonist triggering instead of hCG triggering, freeze-all cycles, and personalized OS protocols can help to reduce the risk of OHSS and improve clinical outcomes. However, further research is needed to determine the optimal approach for individual patients based on their specific risk factors and treatment goals [28].

## Preclinical and laboratory outcomes of ovarian stimulation

Preclinical and laboratory outcomes of OS are also important considerations, as they are associated with clinical outcomes. The number of oocytes retrieved is a key laboratory outcome, as it reflects the efficiency of OS. The number of mature oocytes, defined as those that have reached the metaphase II stage, is another important laboratory outcome, as it is associated with improved pregnancy outcomes [29, 30].

The number of transferable embryos is another important preclinical outcome, as it reflects the quality of the embryos and the likelihood of success. The number of euploid embryos resulting from one stimulation, as assessed by pre-implantation genetic testing for aneuploidy (PGT-A), is also an important laboratory outcome, as it is associated with higher success rates [31]. Although PGT-A is employed to improve IVF outcomes by selecting euploid embryos, its use remains controversial. Critics argue that PGT-A may not significantly increase the chances of a live birth in all patient populations and can lead to the discarding of potentially viable embryos [32]. Ongoing research and debate continue to refine the application and understanding of PGT-A in clinical practice.

Recent studies have also explored the potential for new laboratory techniques, such as time-lapse imaging and metabolomics, as well as artificial intelligence (AI) to improve the selection of viable embryos and increase success rates. Time-lapse imaging allows continuous monitoring of embryo development, which can provide additional information about embryo quality and improve the selection of viable embryos [33–35]. Metabolomics involves the analysis of the metabolites produced by the embryo during development, which can provide insights into the quality and viability of the embryo [36].

Recent advances in laboratory techniques have significantly improved the selection of viable embryos during IVF treatments. One such technique is next-generation sequencing (NGS), which allows comprehensive analysis of the genetic makeup of embryos. NGS can identify chromosomal abnormalities and detect genetic mutations that could impact embryo viability, increasing the chances of selecting the healthiest embryos for transfer [37].

AI is another promising tool for improving embryo selection: it can analyze large datasets of patient and embryo characteristics, as well as time-lapse imaging data, to develop algorithms that accurately predict the likelihood of implantation and live birth. These algorithms can be used to select the most viable embryos for transfer, leading to improved success rates [38].

The impact of laboratory outcomes on patient outcomes, such as implantation and live birth rates, is well established. The number of mature oocytes, number of transferable embryos, and number of euploid embryos resulting from one stimulation are all associated with improved pregnancy outcomes and higher success rates [31].

Besides laboratory outcomes, other patient factors such as age, BMI, and ovarian reserve also play a crucial role in IVF success rates. By incorporating these factors along with laboratory outcomes, clinicians can more accurately predict the likelihood of successful outcomes for individual patients [29].

Overall, continued research and development of laboratory techniques, including NGS and AI, will continue to improve embryo selection and increase IVF success rates. Incorporating these laboratory outcomes alongside patient factors will help clinicians tailor treatment plans to individual patients and improve overall success rates.

## Indicators of successful ovarian stimulation

The most commonly used indicators of successful OS are the number of oocytes retrieved, fertilization rate, and embryo quality. The number of oocytes retrieved is a key indicator of ovarian response and is associated with the chances of pregnancy and live birth. However, the relationship between number of oocytes retrieved and pregnancy outcomes is not always straightforward, as IVF success also depends on other factors such as maternal age and embryo quality [29].

Fertilization rate, defined as the percentage of oocytes that fertilize after insemination, is another commonly used indicator of successful OS. Fertilization rate is associated with embryo quality and pregnancy outcomes, but also has limitations as a predictor of success. Embryo quality, assessed by morphological criteria and chromosomal analysis, is another important indicator of successful OS. High-quality embryos are associated with a higher chance of implantation and live birth [38].

Recent studies have explored alternative indicators of successful OS, such as number of mature oocytes and number of top-quality embryos. The number of mature oocytes has been proposed as a more accurate measure of ovarian response and is associated with improved pregnancy outcomes [29, 39, 40]. However, while traditional indicators such as the number of oocytes retrieved or the fertilization rate may provide some indication of the success of OS, they do not always accurately reflect the chances of achieving a live birth: CLBR is a more comprehensive indicator. It takes into account the use of all fresh and frozen embryos derived from a single OS and provides a more realistic estimation of the chances of achieving a live birth. Therefore, it should be considered as the primary indicator of success in IVF treatments [41]. CLBR is defined as the probability of achieving a live birth from a single OS cycle with all the obtained embryos being transferred until a live birth is achieved [1]. By calculating the CLBR over multiple embryo transfers, the chance of achieving a live birth can be estimated more accurately. Several studies have reported the advantages of using CLBR as an indicator of OS success in IVF treatments [22, 39, 42].

CLBR provides a more comprehensive assessment of the effectiveness of IVF treatments, as it takes into account the cumulative success rates of multiple cycles of treatment. This approach acknowledges that IVF treatments do not always result in a live birth in the first cycle, but the chances of success may increase with subsequent cycles [22, 43]. The use of CLBR as the primary indicator of success in IVF treatments has significant implications for both patients and clinicians. For patients, it provides a more realistic and accurate assessment of the chances of achieving a live birth after multiple cycles of treatment, and helps them to make informed decisions about their treatment options. For clinicians, it highlights the importance of optimizing treatment protocols and strategies to increase the chances of success over multiple cycles of treatment [22].

Despite the advantages of using CLBR, some challenges remain in its implementation. One issue is variability in the number of cycles that patients undergo. Patients may undergo different numbers of cycles, depending on their response to OS or financial constraints. Another variable is the definition of live birth. Studies use different criteria for defining live birth, which can affect the calculation of CLBR [44].

Despite these challenges, the use of CLBR as an indicator of OS success in IVF treatments is gaining acceptance. The European Society of Human Reproduction and Embryology (ESHRE) has recommended the use of CLBR as a primary outcome measure for clinical trials in IVF treatments [1]. The American Society for Reproductive Medicine (ASRM) has also recognized the importance of CLBR as an indicator of IVF success [45].

One important consideration in the context of failed IVF treatments is the phenomenon of drop-out and treatment discontinuation. Research has shown that a significant proportion of couples who start IVF treatment do not complete all the cycles recommended by their doctors. One study found that up to 25% of patients drop out after their first cycle of IVF, and up to 50% after three cycles [46]. This can have important implications for patients’ chances of success, as well as for their mental and emotional well-being.

Several factors have been identified as contributing to drop-out and treatment discontinuation, including financial constraints, physical and emotional burdens of treatment, and lack of social support [47]. It is important for healthcare providers to be aware of these factors and work with patients to address them as early as possible, to improve the likelihood of treatment completion.

Furthermore, research has shown that patients who discontinue treatment after failed IVF cycles could be at increased risk of depression and anxiety [48]. This underscores the importance of providing psychological support to patients throughout the IVF process, particularly in the aftermath of treatment failure.

Thus, considering all these aspects related to drop-out and treatment discontinuation, focusing on providing the best treatment to achieve the optimal cumulative outcome based on patient’s profile should be the norm during IVF treatments. This would decrease the risk of a patient not having their baby due to the burden of failed treatments.

Recent advancements suggest the inclusion of additional markers such as the Follicular Output RaTe (FORT) index, Follicle-to-Oocyte Index (FOI), and the Ovarian Sensitivity Index (OSI) to provide a more comprehensive assessment of ovarian response to stimulation [49, 50].

FORT is a marker that calculates the ratio of pre-ovulatory follicles to small antral follicles visible at the start of stimulation, gives insights into the ovarian responsiveness to gonadotropins, and has been linked with outcomes of IVF cycles [51, 52]. FORT is increasingly recognized as an important marker of ovarian response. It has been demonstrated that FORT could predict the number of oocytes retrieved, linking higher FORT values with better outcomes in IVF treatments. This suggests that FORT could be used to tailor stimulation protocols more effectively, reducing both the time and cost of treatments by optimizing drug dosages early in the stimulation phase [53].

OSI is defined as the total dose of gonadotropins used divided by the number of oocytes retrieved, inversely reflecting the ovarian sensitivity to stimulation, and it serves as a gauge for the efficiency of gonadotropins used during ovarian stimulation [54, 55]; it may also predict the live birth chances after IVF in infertile patients [56]. By measuring the total amount of gonadotropins required to retrieve each oocyte, OSI provides a direct measure of ovarian responsiveness, and it can help identify patients who may require adjusted dosages, potentially reducing the risk of OHSS and improving the overall safety of the IVF process [54].

The FOI has emerged as a potential clinical performance indicator, providing a direct measure of the efficiency of ovarian stimulation in producing viable oocytes for IVF procedures [57]. The FOI can not only help predict successful outcomes, but also enhance routine clinical practice by offering a standardized measure that can guide the customization of treatment plans. The adoption of FOI in clinical settings could significantly improve the strategic planning of ovarian stimulation, ensuring higher efficiency and outcome prediction [50, 58].

These markers collectively represent a shift towards a more nuanced understanding of ovarian stimulation, emphasizing the need for personalized approaches based on detailed phenotypic and dynamic response assessments. Incorporating these indices into clinical practice could revolutionize our predictive capabilities and improve treatment outcomes.

## Future directions

Although current measures of ovarian stimulation success are effective, there is still room for improvement. Continued research is needed to identify new indicators of success, such as alternative laboratory measures and clinical outcomes. The use of AI and machine learning may also hold promise for improving the accuracy of OS prediction and success rates [59–61] . The integration of AI in assessing embryo morphokinetics opens a new frontier in embryo selection. AI algorithms, trained on vast datasets of embryo development images, can predict embryonic developmental potential more accurately than traditional methods. AI can analyze different parameters to select embryos with the highest likelihood of successful implantation and live birth. Recent studies have shown that AI-assisted embryo selection increases the rates of pregnancy and live birth compared to conventional methods [33]. These advancements underscore the potential of AI as a transformative tool in optimizing IVF outcomes.

Further studies are also needed to evaluate the long-term outcomes of IVF treatments, including the health and development of resultant offspring. The effects of OS on the epigenetic and developmental programming of offspring are currently under investigation [62, 63]. Finally, efforts should continue to improve the safety of OS protocols, including prevention of OHSS and the use of more patient-friendly protocols. The development of personalized protocols based on individual patient characteristics, such as age and ovarian reserve, may also improve safety and success rates. Follitropin delta, a new recombinant FSH preparation with a unique individualized dosing algorithm, uses an algorithm to calculate the starting dose of follitropin delta based on serum AMH levels and body weight, trying to ensure a more personalized approach to ovarian stimulation. Studies have demonstrated its effectiveness in reducing the risk of OHSS without compromising the outcomes of IVF treatments [64]. However, there are concerns related to the studies that have compared the clinical outcomes of follitropin delta to alfa because, in general, these studies do not use individualization of starting doses in the groups using the follitropin alfa [65]. Thus, the actual efficacy of this new gonadotropin is yet to be confirmed.

## Conclusion

In conclusion, ovarian stimulation is a critical component of IVF treatment and its success can be defined by a range of clinical, preclinical, and laboratory outcomes, as well as safety outcomes. The number of oocytes retrieved is a key indicator of ovarian response, but it should be considered alongside other indicators such as the number of mature oocytes, number of transferable embryos, and number of euploid embryos resulting from one stimulation. Other factors that can affect OS outcomes, such as age, BMI, ovarian reserve, and type of infertility, should also be considered. Safety outcomes, such as risk of OHSS, should be minimized through appropriate dosing and medication protocols. Preclinical and laboratory outcomes, such as the number of transferable embryos and the results of PGT-A, are important indicators of success and can be improved through new laboratory techniques such as time-lapse imaging and metabolomics.

By taking account of a range of indicators and outcomes, clinicians and researchers can more accurately define and measure successful OS in IVF treatments. Incorporating advanced markers of ovarian responsiveness and understanding the influence of clinical variants are crucial steps toward optimizing IVF treatments. These measures not only promise higher success rates by tailoring approaches to individual needs but also significantly reduce the physical and psychological burden on patients. As the field of reproductive medicine continues to evolve, embracing these innovations will be key to improving the effectiveness and safety of fertility treatments. The integration of comprehensive genomic data into IVF protocols offers a promising avenue to refine treatment strategies further. This approach not only enhances the precision of ovarian stimulation but also contributes to a broader understanding of fertility and reproductive health.

Continued research is needed to identify new indicators of success, improve safety protocols, and evaluate long-term outcomes of IVF treatments.

## Data Availability

Not applicable.

## Abbreviations

AFC: antral follicle count
AI: artificial intelligence
AMH: anti-Müllerian hormone
ASRM: American Society for Reproductive Medicine
BMI: body mass index
CLBR: cumulative live birth rate
DET: deferred embryo transfer
ESHRE: European Society of Human Reproduction and Embryology
FSH[R]: follicle-stimulating hormone [receptor
GnRH: gonadotropin-releasing hormone
hCG: human chorionic gonadotropin
IVF: in vitro fertilization
NGS: next-generation sequencing
OHSS: ovarian hyperstimulation syndrome
OS: ovarian stimulation
PGT-A: preimplantation genetic testing for aneuploidy
